# Supplementary material for: Methods and Challenges of Using the Greater Wax Moth (Galleria mellonella) as a Model Organism in Antimicrobial Compound Discovery
Source: Microorganisms. 2019 Mar 19;7(3):85. doi: 10.3390/microorganisms7030085 (PMC6463077; doi:10.3390/microorganisms7030085)
Supplement: Supplementary file 1 [file microorganisms-07-00085-s001.pdf]

*microorganisms*

*Article*

# Methods and Challenges of Using the Greater Wax Moth (*Galleria mellonella*) as a Model Organism in Antimicrobial Compound Discovery

**Athina Andrea [1], Karen Angeliki Krogfelt [1,2] and Håvard Jenssen[1,*]**

[1] Department of Science and Environment, Roskilde University, 4000 Roskilde, Denmark; atan@ruc.dk (A.A.); kak@ssi.dk (K.A.K.)
[2] Virus and Microbiological Special Diagnostics, Statens Serum Institut, 2300 Copenhagen, Denmark
* Correspondence: jenssen@ruc.dk

**Text S1. Selection of appropriate-for-testing larva.**

In the majority of publications where *G. mellonella* model is studied, the larvae are either purchased from commercial sources or bred in house in research facilities. Commercially available *G. mellonella* larvae are relatively inexpensive and easily accessible, because they are commonly sold as food for pet-reptiles. For the larvae from commercial sources, breeding conditions are in many cases unknown with regard to food composition, use of antimicrobials or growth promoters, temperature etc., parameters that might have a significant impact on the physiology of the larvae. For the cases where *G. mellonella* is in-house bred, the conditions are more controlled. However, breeding larvae systematically in a large scale, is not an easy task, requiring experience, dedicated lab space and personnel. In our lab, larvae are purchased from local vendor in Copenhagen, and arrive to Roskilde University by post. Larvae are in wood chips (Figure 1), which are a food source for the larvae. Wood chips must be removed since food consumption influences the immunity and it has been reported that food deprivation of *G. mellonella* larvae leads to reductions in cellular and immune response [1]. Upon arrival, larvae can be in a variety of conditions, and dead larvae are often found. Therefore, it is important that the healthy larva are selected upon arrival and stored in appropriate conditions until used for testing. Dead and sick larvae are removed just so they do not compromise the health of the other larvae. Dead and sick larvae can be easily recognized and discarded. Unhealthy larvae appear to be of darker color due to melanization reaction or have dark spots at their body (Figure 1). Larvae must be examined carefully, both at dorsal and ventral body sides. Emphasis should be also given to the prolegs, as healthy-looking larvae might still have clogged prolegs, which will hinder the injection procedure. Healthy larvae are challenged further by turning them to their back and see if they can quickly turn. To minimize the variability we recommend to use larvae from the same provider, so the genetic background of the larvae and the breeding conditions are the same. Finally, we recommend to acclimatize the selected healthy larvae to 37°C one day before the experiments, as occasionally some of them die (Figure S1, part 8).

One of the first things one should do when running experiments with larvae is to figure out which weight range should be employed. To our experience, very small larvae (less than 200mg) should be avoided because the injection procedure becomes more challenging technically, and also because the small size might indicate an earlier developmental stage. Choice of weight range is crucial as the responses of the animals to infection or test compounds can be influenced by the size. Dosing reproducibility can be very challenging when larva weight is changed from experiment to experiment. The same dose of bacteria can be lethal for larva of certain weight, but not for bigger larva (Figure 2). In order to use as many larvae from each batch as possible, an estimation of the distribution of the larva weight should be performed. An example can be seen in Figure S2. In order to use as many larvae as possible from each batch, we recommend to pick up the most abundant weight range. In our experience, there is variation between the different vendors, but larvae weight distribution from the same vendor is quite standard.

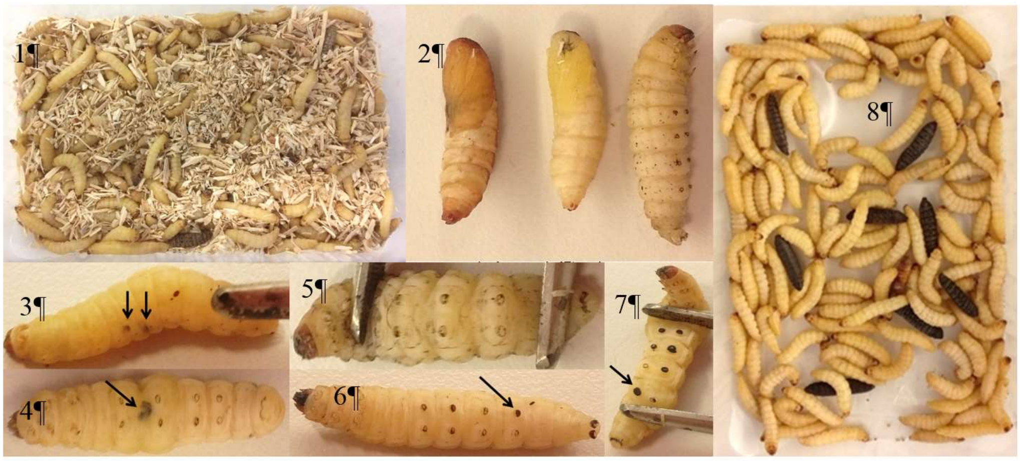


**Figure S1.** Selection of *G. mellonella* larvae. (1). A typical batch of *G. mellonella* larvae, purchased from local vendor in Copenhagen and received at Roskilde University, by post. The larvae are delivered in wood chips. (2). Usually larvae at the early (right larva) or late pupation (left and middle larvae) stage are encountered in the received batch, which must be removed and excluded from experiments. Early pupation signs are akinesia, curvature behind the head and degeneration of prolegs. (3–7). Examples of larvae unsuitable for experiments, due to signs of sickness (some indicated by arrows). Melanisation can be observed both dorsal and ventral, epidermally or deeper in the larva tissues. Clogged prolegs in 6 and 7 hinder the injection, therefore larvae must be discarded. (8). Pre-incubation of healthy larvae at 37 °C for around 24 hours reveals more unsuitable larvae (dead larvae are black).

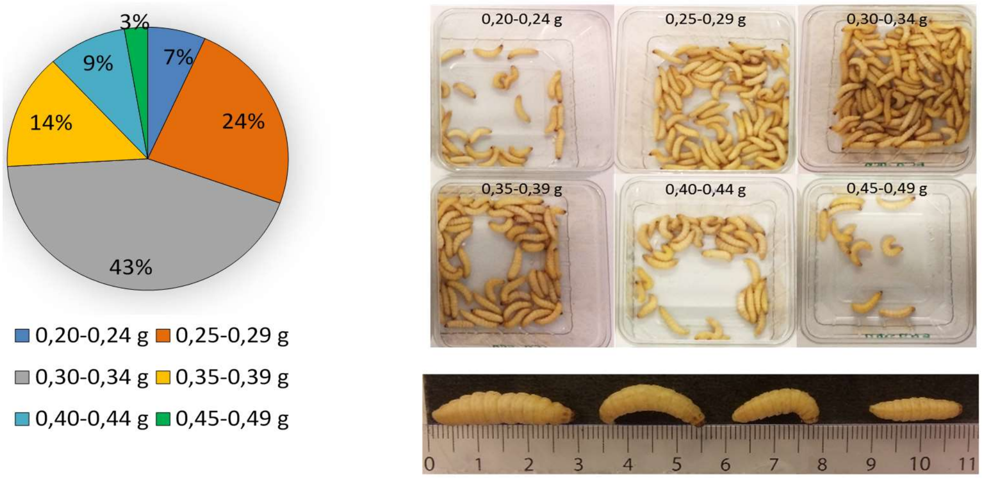


**Figure S2.** Size variability among a larva batch. Larvae were purchased and distributed according to weight. Approximately 1000 larvae were purchased and healthy larvae were selected, and distributed according to weight.

## References

1. 1. Banville, N.; Browne,N.; Kavanagh, K. Effect of nutrient deprivation on the susceptibility of Galleria mellonellalarvae to infection. *Virulence*, **2014**, *6*, 497–503.

© 2019 by the authors. Submitted for possible open access publication under the terms and conditions of the Creative Commons Attribution (CC BY) license (http://creativecommons.org/licenses/by/4.0/).
